# BK Channels Regulate Spontaneous Action Potential Rhythmicity in the Suprachiasmatic Nucleus

**DOI:** 10.1371/journal.pone.0003884

**Published:** 2008-12-08

**Authors:** Jack Kent, Andrea L. Meredith

**Affiliations:** Department of Physiology, University of Maryland School of Medicine, Baltimore, Maryland, United States of America; Yale School of Medicine, United States of America

## Abstract

**Background:**

Circadian (∼24 hr) rhythms are generated by the central pacemaker localized to the suprachiasmatic nucleus (SCN) of the hypothalamus. Although the basis for intrinsic rhythmicity is generally understood to rely on transcription factors encoded by “clock genes”, less is known about the daily regulation of SCN neuronal activity patterns that communicate a circadian time signal to downstream behaviors and physiological systems. Action potentials in the SCN are necessary for the circadian timing of behavior, and individual SCN neurons modulate their spontaneous firing rate (SFR) over the daily cycle, suggesting that the circadian patterning of neuronal activity is necessary for normal behavioral rhythm expression. The BK K^+^ channel plays an important role in suppressing spontaneous firing at night in SCN neurons. Deletion of the *Kcnma1* gene, encoding the BK channel, causes degradation of circadian behavioral and physiological rhythms.

**Methodology/Principal Findings:**

To test the hypothesis that loss of robust behavioral rhythmicity in *Kcnma1^−/−^* mice is due to the disruption of SFR rhythms in the SCN, we used multi-electrode arrays to record extracellular action potentials from acute wild-type (WT) and *Kcnma1^−/−^* slices. Patterns of activity in the SCN were tracked simultaneously for up to 3 days, and the phase, period, and synchronization of SFR rhythms were examined. Loss of BK channels increased arrhythmicity but also altered the amplitude and period of rhythmic activity. Unexpectedly, *Kcnma1^−/−^* SCNs showed increased variability in the timing of the daily SFR peak.

**Conclusions/Significance:**

These results suggest that BK channels regulate multiple aspects of the circadian patterning of neuronal activity in the SCN. In addition, these data illustrate the characteristics of a disrupted SCN rhythm downstream of clock gene-mediated timekeeping and its relationship to behavioral rhythms.

## Introduction

In mammals, the suprachiasmatic nucleus (SCN) of the hypothalamus is the primary circadian pacemaker, controlling daily rhythms in physiology and behavior. The mechanism of intrinsic rhythmicity in the SCN was discovered when several “clock genes” that control circadian pacemaker function were identified in flies and mice [1]. These genes encode transcriptional activators (CLOCK and BMAL1), and repressors, (PER1-3, CRY1-2, and REV-ERBα), and they comprise a transcriptional-translational feedback loop that occurs with ∼24 hr kinetics in the absence of light input to the SCN [2], [3]. Although the basis for time-keeping within individual SCN neurons is generally understood to rely on these core clock genes, this alone does not reveal how behavioral rhythms are controlled. The essential links between the generation of neuronal clock gene rhythms and translation of a consolidated SCN time signal to behaviors are unknown.

One link between the molecular clockwork and circadian behavioral rhythms is expressed at the level of neuronal activity. Action potentials in the SCN are necessary for normal circadian timing of behavior [4], and spontaneous action potentials in the SCN show diurnal patterning. During the day neurons are more active, increasing their activity to 8–10 Hz at the midday peak. At night, activity is suppressed to <2 Hz on average, with many neurons remaining silent [5]–[9]. These oscillations in spontaneous firing rate (SFR) continue even in the absence of light input to the pacemaker [9], when time-keeping relies on the function of the core clock genes [1]. Consequently, the daily SFR rhythm reflects the intrinsic periodicity of clock gene expression in the SCN, and mutations alter SFR rhythms in individual SCN neurons in a way that reflects the primary pacemaker alteration and behavioral outputs. For example, the *tau* (casein kinase 1ε) mutation in hamsters shortens the period of SFR rhythms in a dose-dependent manner, consistent with shorter periods in wheel-running activity in *tau* animals [10]. Similarly, *Clock* heterozygotes have lengthened SFR and behavioral rhythms, while homozygotes are arrhythmic [11], [12]. *Cry1/2* double mutants also show SFR and behavioral arrhythmicity [13]. These findings suggest that clock gene rhythms are translated into daily patterns of action potential activity in the SCN.

Beyond clock genes, several ionic currents in the SCN have been recently identified that regulate the day-night difference in neuronal firing rate and thus shape SFR rhythmicity. At night, when neurons are silent or fire at low frequencies, the BK Ca^2+^-activated K^+^ current is the only specific current identified so far that regulates SFR. Block of BK channels or deletion of *Kcnma1*, the gene encoding the pore-forming subunit of the BK channel, increases nighttime firing in SCN neurons, although not completely abolishing the day-night difference in SFR [14], [15]. Conversely, during the day when firing rate is higher, blocking the fast delayed rectifier (FDR) K^+^, voltage-gated Ca^2+^, or an unidentified (non-BK) Ca^2+^-activated K^+^ current all decrease SFR [16]–[18]. The influence of Ca^2+^, FDR, and BK currents during discrete time windows appears to be due in part to diurnal variation in their current magnitudes [15]–[17].

The molecular constituents for these currents, with the exception of BK, have not been identified, leaving the behavioral significance of their roles in regulating the SFR unknown. However, deletion of the BK channel causes disruption of circadian behavioral rhythms in *Kcnma1^−/−^* mice and *slowpoke* flies [14], [19], providing the opportunity to use this mutation in mouse to study the relationship between how a single ionic current regulates SCN-level rhythms and how this current regulates behavioral timing. Consequently, to understand how changing neuronal firing in the SCN causes a change in behavior, we examined the circadian parameters of SFR rhythms in greater detail by long-term multi-electrode array recordings. We hypothesized that loss of robust circadian rhythmicity in *Kcnma1^−/−^* mice was generated at the level of the SCN. Recordings from *Kcnma1^−/−^* SCN slices show alterations in SFR rhythmicity and phase variability compared to wild-type (WT). These data support a critical role for BK channels in the SCN to generate of rhythmicity, in addition to their role in regulating firing frequency.

## Methods

### Animals

All procedures involving mice were conducted in accordance to the University of Maryland Institutional Animal Care and Use Guidelines. WT and *Kcnma1^−/−^* mice were maintained on an inbred FVBN/J background and genotyped as described previously [20]. All mice were group housed on a standard 12∶12 light∶dark (LD) cycle until brain harvest.

### Acute SCN slice preparation

Mice were anesthetized with isoflurane at lights on (zeitgeber time, ZT0), and brains were rapidly harvested into ice cold artificial cerebrospinal fluid (ACSF), in mM: 119 NaCl, 1.3 MgSO_4_, 26 NaHCO_3_, 1 NaH_2_PO_4_, 5.3 KCl, 2.5 CaCl_2_, 20 glucose. Acute coronal slices were cut at 300 µm on a VT1000S vibratome (Leica, Wetzlar, Germany) at 3°C in ACSF. Slices containing SCN were recovered for 1–2 hrs at 36°C in oxygenated ACSF in a submerged recovery chamber (BSK-AM, Sci Sys, Inc., Mississauga, Canada). Multi-electrode array P210A probes (Alpha MED Sciences, Osaka, Japan), with 64 electrodes spaced 100 µm apart, were initially treated with 0.1% polyethylenimine solution in 25 mM borate buffer overnight to decrease hydrophobicity and facilitate sticking. Slices were placed onto probes, positioning the SCN directly over the 8×8 electrode grid, and weighted. Oxygenated ACSF plus 0.16 mg/ml gentamicin (Sigma) was continuously perfused through an interface chamber (Med 64, Alpha MED Sciences) at 1–2 ml/min. The temperature was maintained at 30–32°C for the duration of the recording. Tetrodotoxin (Sigma) stocks were prepared in 1 mg/ml BSA.

### Extracellular action potential recordings

Signals from all electrodes on the MEA probe were collected simultaneously with the Med CO2P connector headstage and 64-channel integrated amplifier (Med 64, Alpha MED Sciences). Ten second data samples were collected every 15 minutes at 20 kHz in Conductor v3.1f (Alpha MED Sciences). Channels within the SCN were identified visually, and spontaneous extracellular action potentials recordings from these SCN channels were discriminated offline using threshold-based event counting. Thresholds were typically set at 1.5-2X the baseline noise level, with typical signal amplitude between 20 and 75 µV. Single unit discrimination was not routinely possible in these experiments; however, we estimated that recordings at each channel were between one and ten neurons based on histogram analysis of event amplitude and individual firing rates derived from cell-attached and intracellular recordings [14]. In a few experiments, the baseline noise spontaneously increased during the course of the experiment and thresholds were reset. Peak-to-trough (PT) ratios were not calculated from any data set where the threshold was reset.

### Analysis of SFR rhythms and statistics

The SFR activity from each channel located within the SCN on the MEA probe was classified as rhythmic (R), if it had a daily peak (defined as the peak value being at least 1 Hz greater than the trough value and one peak per ∼24 hr cycle), or arrhythmic (AR), if the recorded activity failed to meet these criteria. After categorizing channels as R or AR, the time of the peak was calculated by smoothing the data with a 2 hr moving window average. The daily SFR peak, usually ∼CT6 (circadian time) in WT, was used as the phase reference point for calculating the period of SFR rhythms from consecutive cycles. PT ratios were calculated as (P-T)/P, where P = the average SFR from CT 5–7 (peak) and T = the average SFR from CT 12–14 (trough). These times correspond to the maximum differences in firing rate from WT slices. In some cases, the peaks of *Kcnma1^−/−^* SFR rhythms were closer to CT 12–14 than the expected peak at CT 6, yielding a negative PT value. For each cycle individually, SFR rhythm parameters (time of phase peak, period, PT ratio) were compared between WT and *Kcnma1^−/−^* (average±standard error of the mean, s.e.m., or standard deviation, s.d.). Significance of comparison between genotypes was determined by an unpaired, two-tailed *t*-test, *p*<0.05. Significance of rhythmic versus arrhythmic recordings was determined over 3 cycles by χ^2^ test with two degrees of freedom (*p*<0.05).

## Results

### Circadian rhythmicity in acute SCN slices

**Figure 1:**
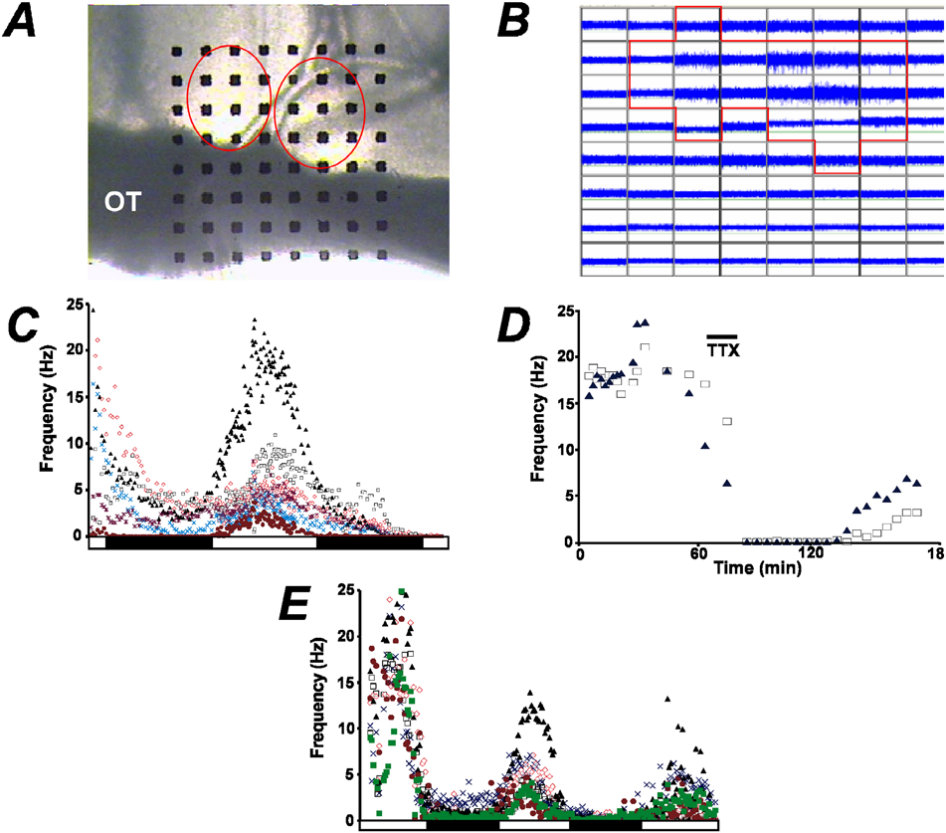
Multi-electrode array recordings of SFR rhythms from SCN. *A,* Acute SCN slice (300 µm) on probe. Circles outline SCN. OT = optic tract. Electrode spacing = 100 µm. *B,* Oscilloscope view from slice in (*A*) showing spontaneous activity at each electrode (channel) on the probe. Red outlines channels within the SCN. 1 box = 2.5 sec/100 µV. *C,* Six active channels within the SCN showing a robust circadian regulation of the SFR over 40 hr. Light and dark bars indicate the prior entrainment of the animal before slice harvest. *D,* Perfusion of 1 µM tetrodotoxin (TTX) blocks spontaneous action potentials (2 channels shown). Washout was started 5 min after TTX addition. *E,* Synchronized rhythms over 3 circadian SFR peaks.

To understand the early steps in circadian rhythm generation, we analyzed SFR rhythms in acute slices harvested from adult animals. In this study, we measured extracellular action potentials over 3 days using a planar 64-channel multi-electrode array. In an acute slice, up to 20 electrodes on the probe were within the average sized adult SCN (Fig. 1*A*). Generally 5–10 electrodes showed a robust signal-to-noise ratio suitable for analysis (Fig. 1*B*). Most of these recordings were determined to be multi-unit, estimated by event amplitude analysis, derived from the activity of <10 neurons. Spontaneous activity from these small populations of neurons showed entrainment to the prior LD cycle of the animal with a synchronous peak in the middle of the day (Fig. 1*C*). The daily patterns of activity were consistent with previous studies using other recording methodology in acute slices [21]–[24]. To verify the nature of spontaneous activity, action potentials were blocked by tetrodotoxin (TTX), a selective blocker of voltage-gated Na^+^ channels (Fig. 1*D*). Over the 3 day recording period, the amplitude of the SFR rhythm decreased, presumably due to slice remodeling from perfusion flow and death of neurons. However, circadian patterns of activity throughout the life of the slice were observed during the entire recording period (Fig. 1*E*). The circadian period and amplitude could be determined for some SFR rhythms with χ^2^ periodogram analysis (data not shown), which is used to analyze behavioral rhythms [25]. Unfortunately, we found the systematic analysis of SFR rhythms using these methods problematic due to the low number of circadian cycles. However, the circadian period could be determined from subtracting the daily peaks of SFR rhythms (see next section). The ability to measure the circadian characteristics of SFR rhythms in acute slices on the MEA motivated our investigation of an SCN-level phenotype in WT and BK null (*Kcnma1^−/−^*) mice.

### Loss of BK channels degrades SFR rhythmicity

**Figure 2:**
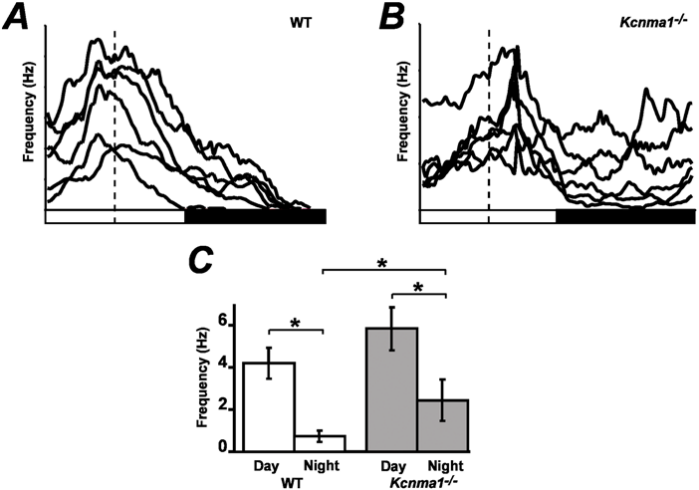
SFR rhythms from WT and *Kcnma1^−/−^* SCNs. *A* and *B,* MEA recordings from 6 channels on cycle 2 within a WT (*A*) and a *Kcnma1^−/−^* (*B*) SCN over 24 hr. Data were smoothed by applying a 1-hr moving window average and plotted to maximize any day-night differences in firing rate. Dotted lines denote CT6 (6 hours after lights on for the animal prior to slice harvest), the daytime peak for SFR. *C,* Individual firing frequencies from cycle 2. The daytime peak (CT5-7) and trough (CT12–14) SFR values were from 5 WT (*n* = 32 channels) and 4 *Kcnma1^−/−^* (*n* = 30 channels) slices±s.e.m. *p<0.05.

We previously showed that *Kcnma1^−/−^* mice had decreased circadian behavioral rhythm amplitude, which correlated with hyperactive firing of individual SCN neurons at night [14]. However, the inability to hold single cells over circadian time frames restricted the ability to measure the parameters of circadian rhythmicity in a defined neuronal population over successive cycles. In this study, to determine whether the circadian pattern of neuronal activity was altered in the absence of BK currents, we measured SFR rhythms from WT and *Kcnma1^−/−^* slices. In the first cycle after slice harvest, SFR activity from all WT SCNs was rhythmic, showing a day-night difference in SFR (Fig. 2*A* and Table 1). In contrast, on the first cycle after slice preparation from *Kcnma1^−/−^* SCNs, a third of the recordings showed arrhythmic SFRs (Fig. 2*A* and Table 1). Some arrhythmicity did develop in WT SCNs over the 3 day recording period (∼10%); however, the proportion of arrhythmicity in *Kcnma1^−/−^* slices remained consistently higher than WT for each cycle (21–37%, *p*<0.5, Table 1). Furthermore, the number of arrhythmic recordings from *Kcnma1^−/−^* SCNs was relatively constant over the 3-day recording period, showing that the arrhythmicity was already present at the time of slice preparation and did not develop *de novo* during the recording period. The increased arrhythmicity in *Kcnma1^−/−^* recordings suggests that BK channels are necessary for regulating the day-night difference in SFR and shaping daily spontaneous activity rhythms in the SCN.

**Table 1:** Analysis of WT and *Kcnma1^−/−^* SFR rhythms.

	Day 1	Day 2	Day 3
**WT**	*n* = 39	*n* = 57	*n* = 11
	R = 39 (6.19±0.95 hr)§	R = 50 (5.70±1.15 hr)#	R = 10 (5.43±0.41 hr)§
	AR = 0 (0%)	AR = 7 (12.3%)	AR = 1 (9.1%)
	PT = 0.90±0.17*	PT = 0.87±0.15†	
	*n* = 23	*n* = 30	
***Kcnma1^−/−^***	*n* = 30	*n* = 38	*n* = 12
	R = 19 (6.78±1.62 hr)	R = 30 (6.83±1.55 hr)#	R = 8 (4.91±2.04 hr)
	AR = 11 (36.7%)	AR = 8 (21.1%)	AR = 4 (33.3%)
	PT = 0.52±0.31*	PT = 0.54±0.40†	
	*n* = 10	*n* = 29	

Spontaneous activity was analyzed from channels within the SCN that had activity >1 Hz. R = the number of rhythmic channels (in parentheses: average time of the SFR peak±s.d.). AR = the number of arrhythmic channels (% of total). *N*-values are presented as the number of rhythmic and arrhythmic channels from 5 WT and 4 *Kcnma1^−/−^* slices. Separate *n*-values (bottom of cell) are presented for peak-to-trough (PT) ratios because these values could not be extracted from every recording due to noise problems. Peak and trough frequencies were determined by averaging two hours of continuous raw data from rhythmic channels at CT 5–7 and CT 12–14, respectively. The PT ratio was calculated for each channel as (P–T)/P and is presented as the average±s.d. ^*, #, §, †^ *p*<0.05.

To determine whether the remaining rhythmic activity was also altered in the absence of BK channels, we measured the peak-to-trough (PT) ratios of WT and *Kcnma1^−/−^* SFR rhythms, an indicator of the robustness of the circadian variation in SFR. Of the channels that exhibited rhythmic activity, neuronal firing rates were averaged during the day at CT5-7 (peak) and at night, CT12–14 (trough), to determine PT ratios. WT SCNs had high neuronal activity during the day (4.2±0.72 Hz, *n* = 32) with a significant decrease in SFR at night (0.72±0.28 Hz, Fig. 2*C*). These recordings exhibited PT ratios closer to 1 (Table 1), as expected for recordings from neuronal populations with a robust diurnal variation in firing rate. In contrast, *Kcnma1^−/−^* PT ratios were 60% lower than WT on average (*p* = 0.0035). The PT ratio from *Kcnma1^−/−^* SCNs was changed largely due to an increase in firing at night (2.4±0.98 Hz, *n* = 30, Fig. 2*C*), although some increase in firing during the day was observed (5.8±1.0 Hz) compared to WT (*p* = 0.06). Consequently, by measuring PT ratios, these data show that even the rhythmic recordings from *Kcnma1^−/−^* SCNs had a weakened circadian variation in SFR. Both the increase in arrhythmic recordings and weaker PT ratios together suggest that BK channels play a general role in maintaining high amplitude circadian rhythms in neuronal activity in the SCN.

### BK channels regulate SFR period and the precise timing of the daily peak

**Figure 3:**
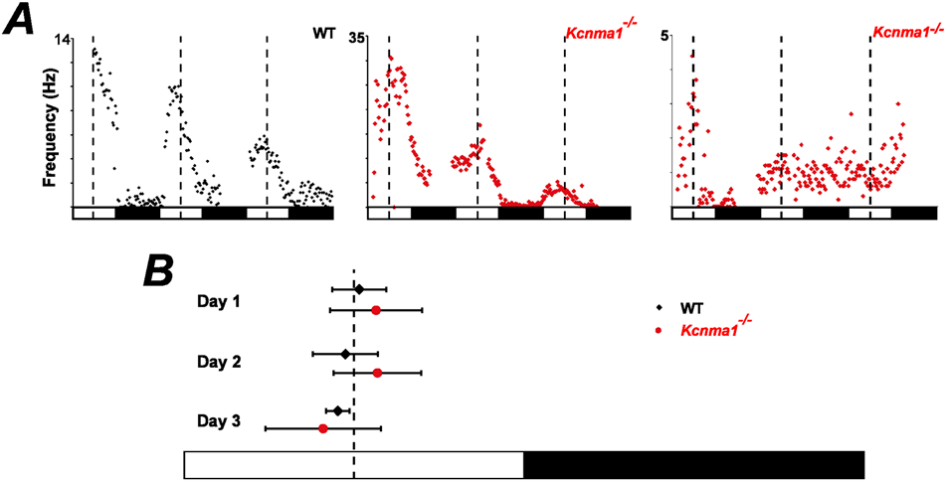
SFR rhythms from 3 circadian cycles from WT and *Kcnma1^−/−^* SCNs. *A,* Representative recordings over 3 cycles from a rhythmic WT (*left*), a rhythmic *Kcnma1^−/−^* (*middle*), and an arrhythmic *Kcnma1^−/−^* (*right*) channel within the SCN. *B,* Distribution of daily peaks for WT and *Kcnma1^−/−^* SFR rhythms (± s.d.) on each day of recording.

The characteristics of a circadian rhythm are represented by the amplitude, period, and phase of activity. *Kcnma1^−/−^* neurons lost their high amplitude variation in spontaneous firing activity over the daily cycle in the SCN compared to WT. However, *Kcnma1^−/−^* firing patterns could change in several ways that ultimately sum to produce a decreased day-night difference in firing rate at the level of the SFR rhythm. First, BK channels could be required to directly determine the day and night firing rates in individual SCN neurons. This hypothesis is supported by the change in PT ratio and increased arrhythmicity in *Kcnma1^−/−^* recordings. Consistent with this, action potential recordings from individual neurons in the SCN do show an increase in the SFR at night in *Kcnma1^−/−^* neurons [14]. Alternately, or in addition, BK channels could regulate the synchronization of neurons, and averaging of out-of-phase SFR rhythms could result in a net reduction in the day-night variation in SFR. Multi-electrode array recordings, where multiple SFR rhythms can be recorded within each SCN, allow the latter model to be tested. Therefore, to address the level of synchronization among the neurons in *Kcnma1^−/−^* SCNs, we analyzed the timing of the daily SFR peak from the population of rhythmic recordings (Fig. 3).

**Figure 4:**
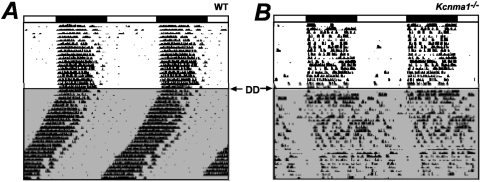
Representative wheel running rhythms from WT and *Kcnma1^−/−^* mice. Mice were housed on a standard 12 hr light: 12 hr dark cycle for 18 days (light and dark bars above actogram), and then placed in constant darkness (DD) for 25 days (shaded bottom part of actogram). Tick marks denote wheel running and actograms are double-plotted to emphasize the behavioral rhythm. *A,* The WT mouse has a circadian period of 23.5 hr in DD, and the χ^2^ circadian amplitude of the behavioral rhythm was 2390. *B,* Representative *Kcnma1^−/−^* actogram (period = 24.2 hr and χ^2^ circadian amplitude = 375).

WT SCNs showed a synchronous peak of activity at midday, with the average peak of the first two cycles at CT5.95 (Fig. 3*A* and Table 1). The prior LD cycle of the intact animal predicts a peak in neuronal activity at CT6. Acute slices from WT animals revealed an interesting phenomenon that mimics behavioral rhythms. The WT phase peak shifted earlier each day (Fig. 3*B*), resulting in an average WT SFR period of 23.62±0.31 hr (± s.e.m.) between cycle 1 and cycle 2. The preparation of an acute slice, which separates the SCN from retinal inputs, thus mimics placement of an intact animal in constant darkness (DD), where the period of behavioral rhythms for WT mice of the same strain background decreases from 24 hrs in LD to ∼23.73 hr in DD (Fig. 4*A* and [14]).

In contrast, *Kcnma1^−/−^* slices had later phase peaks for the first two cycles than WT (30 min–1 hr, Table 1). Overall, the later phase peaks resulted in a slightly longer average period, 24.05±0.44 hr, for *Kcnma1^−/−^* SFR rhythms (*p* = 0.68). Although the difference was not statistically significant between WT and *Kcnma1^−/−^*, this ∼25 min increase in SFR period compared to WT is observed at the level of *Kcnma1^−/−^* behavioral rhythms (Fig. 4*B* and [14]). The current study reveals that even a small change in the behavioral period can be encoded at the level of the SCN.

We did not include the third daily activity peak in period calculations due to the high variability in *Kcnma1^−/−^* recordings. In fact, both the variability for *Kcnma1^−/−^* SFR peaks between recordings from the same slice, as well as across slices, was significantly higher than WT on each cycle. The average intra-slice standard deviation was 1.40 hr in *Kcnma1^−/−^* recordings (*n* = 7 cycles), compared to 0.56 hr for WT (*n* = 10 cycles). Therefore, the intra-slice variability in the absence of BK channels was not higher than the average inter-slice variability within genotypes (average s.d. between slices, WT: 0.69 hr and *Kcnma1^−/−^*: 1.39 hr). The comparison of variability in the daily SFR peak suggests that the timing of the daily peak is less precise in *Kcnma1^−/−^* SCNs compared to WT and also suggests that this observation is not a result of inter-slice variability, but represents a loss of precise synchrony within slices. In contrast, full desynchronization would result in a random distribution of SFR peaks across the circadian cycle. This was not observed in *Kcnma1^−/−^* SCNs, suggesting that BK channels do not play a major role in synchronization.

## Discussion

Here we show the ability to record circadian patterns in neuronal activity from acute adult SCN slices on a multi-electrode array. Compared to recordings from acute SCN slices with metal or glass extracellular electrodes which typically produce only one or two circadian cycles of activity before losing stability of the recording configuration [21]–[24], multi-electrode array recordings of SFR can be used to determine the period from 2 successive activity peaks, providing an average SFR period value for each recording. In addition, steric constraints limit the number of electrodes that can be placed in one SCN simultaneously, while a multi-electrode array probe of 100 µm electrode spacing can conceivably record from up to ∼10 locations per SCN. This capability allows measurement of synchrony from an acute slice over multiple cycles. Although multi-electrode array recordings from organotypic SCN cultures provide the same ability to measure synchrony, slices are prepared from neonates before the eyes open and before behavioral rhythms can be measured.

In this study, we recorded SFR rhythms by multi-electrode array to determine how neural activity rhythms in the SCN were disrupted in animals missing the BK channel, which had previously been shown to be important for regulating SCN firing rate at night and also for generating robust behavioral rhythms [14]. The SCN-level phenotype for *Kcnma1^−/−^* mice includes an increase in arrhythmicity, as well as changes in the parameters of rhythmic SFR activity, measured as a decrease in the circadian variation in firing rate (PT ratio), a decrease in the timing precision of the daily peak, and a ∼25 min lengthening of the average circadian period of the SFR rhythm. The loss of robust SFR rhythmicity in the SCN is overtly similar to the behavioral rhythm in *Kcnma1^−/−^* mice, including a decrease in the circadian amplitude of the SFR rhythm and a slight lengthening of the circadian period. In total, the correlation between the SCN-level and behavioral phenotypes suggests that the daily patterning of neuronal activity in the SCN is a critical regulator of circadian behavior.

These results also clearly substantiate the role for BK channels regulating circadian rhythmicity within the SCN pacemaker, even though they are expressed outside of the SCN as well. It has also been shown that the total daily amount or distribution of wheel activity can affect entrainment and the period of behavioral rhythms, suggesting that locomotor activity can feedback on the SCN pacemaker [26]–[29]. Consequently, the ataxia and hyperactivity of *Kcnma1^−/−^* mice could have confounded the isolation of the circadian behavioral phenotype to the SCN [14]. However, we demonstrate here clear alterations in SCN rhythmicity that closely parallel behavioral rhythms. Furthermore, these data corroborate the placement of BK activity downstream of clock-gene mediated timekeeping in the SCN. Despite the high proportion of arrhythmic neurons revealed by SFR recordings, clock gene expression patterns were not grossly disrupted in the SCNs of *Kcnma1^−/−^* mice [14]. In summary, BK channels control multiple aspects of the SCN-level rhythm and pacemaker output, and the mutant phenotype shows similar complex characteristics at the level of the SCN to circadian behavioral rhythms.

SCN-level rhythmicity in neuronal activity relies on both intrinsic currents that generate the day-night difference in activity, as well as intercellular communication to synchronize the rhythms of individual neurons. We previously demonstrated a role for BK channels in generating the day-night difference in frequency [14]. In this study, by following neuronal firing continuously over longer time scales, we found a previously undetected increase in overall arrhythmicity, as well as a decrease in the robust day-night difference in the firing rate of the residually rhythmic neurons. In addition, the amplitude, phase, and period of the remaining rhythmic SFR activity was altered. Overall, about a third of the neurons in the SCN rely on BK channels to maintain overt rhythmicity, while the residual rhythmic neurons require BK channels for the high amplitude circadian variation or precise timing of their daily activity.

This dual role has also been observed for the SCN-expressed neuropeptide, vasoactive intestinal peptide (VIP). *VIP^−/−^* and *Vipr2^−/−^* SCNs show a 70% decrease in the number of rhythmic neurons, and the residually rhythmic neurons show significant desynchronization and unstable periods [30]. Given the less severe phenotype in *Kcnma1^−/−^* SCNs, it seems unlikely that BK channels transduce a significant component of VIP signaling. Instead, loss of the precise timing of daily SFR peaks suggests that BK channels play a general role in controlling excitability in most neurons in the SCN. Consistent with this, the channels are expressed throughout the SCN [14]. In some cells that express a *Per1-*GFP reporter, the BK current is diurnally modulated, with the highest current density at night when SFR is low [15]. These cells may be strong oscillators that maintain some level of rhythmicity in *Kcnma1^−/−^* SCNs. Alternately, weaker oscillators may lose rhythmicity entirely when BK channels are deleted. The idea of BK channels having different influence over different classes of neurons in the SCN is also supported by electrophysiological data. Some groups did not find evidence for a significant role for BK channels in regulating firing during the day (Fig. 2*C* and [14], [18]). However, *Per1*-GFP expressing neurons appear to more strongly require BK channels to suppress firing during the day [15] than the SCN population as a whole [14]. Whether these data can be reconciled by accounting for procedural differences or indicate a complex role for BK during the day remains to be determined.

Another point that remains to be addressed is what components of the degradation of circadian behavioral rhythms in *Kcnma1^−/−^* mice can be ascribed to the loss of SFR rhythmicity versus just the overall increase in nighttime firing. The generation of robust circadian behavior could rely strongly on both the proportion of rhythmic neurons in the SCN, as well as the waveform of daily neuronal rhythms (amplitude/PT ratio, period, and timing of the peak). In flies, hyperexcitation of the LN_v_s pacemaker neurons through expression of NaChBac, a low threshold slowly inactivating Na^+^ channel, resulted in complex alterations of free-running rhythms [31], suggesting that increasing neuronal activity alone can disrupt circadian behavioral rhythms. Furthermore, in LD conditions, these flies showed a reversed neural activity rhythm leading to increased locomotor behavior at night [32], establishing the idea that the day-night patterning of action potentials in *Drosophila* clock neurons regulates circadian behavior. In contrast, expression of a truncated *Shaw* K^+^ channel in flies, which is also expected to increase neuronal activity, only modestly affects locomotor rhythms [33]. In the mammalian SCN, a direct test of the relative contributions of the number of rhythmic neurons versus the contribution of the rhythmic waveforms for robust circadian behavior remains to be addressed. However, our data here provide a unique window into this issue. WT and *Kcnma1^−/−^* SFR recordings suggest that the combination of losing ∼30% of the rhythmic firing patterns and changing the parameters of the residually rhythmic activity (decreased circadian variation in firing rate, slightly lengthened period, and increased variability of the daily firing peak) is enough to produce a phenotype in circadian behavioral rhythms.
